# *Ex vivo* Coronary Angiography: Safety of Iopromide in Cold Preservation of Pig Hearts

**DOI:** 10.21470/1678-9741-2024-0080

**Published:** 2025-02-11

**Authors:** Maksim O. Zhulkov, Dmitry A. Sirota, Ilya S. Zykov, Olga V. Poveshchenko, Maria A. Surovtseva, Irina A. Kim, Andrey V. Protopopov, Azat K. Sabetov, Khava A. Agaeva, Alexandr G. Makaev, Aleksandr P. Nadeev, Vladislav E. Kliver, Evgeniy E. Kliver, Alexander M. Volkov, Natalya A. Karmadonova, Yaroslav M. Smirnov, Alexey D. Limanskiy, Aleksandra R. Tarkova, Aleksandr M. Chernyavskiy

**Affiliations:** 1 Adult Cardiac Surgery Department, Meshalkin National Medical Research Center, Novosibirsk, Russian Federation; 2 Department of Cardiovascular Surgery, Novosibirsk State Medical University, Novosibirsk, Russian Federation; 3 Cell Technology Laboratory, Research Institute of Clinical and Experimental Lymphology - Branch of the Institute of Cytology and Genetics, Siberian Branch of the Russian Academy of Sciences, Novosibirsk, Russian Federation

**Keywords:** Cardiac Output, Cold Temperature, Coronary Sinus, Creatine Kinase, Myocardial Ischemia, Myocytes, Cardiac, Ischemia, L-Lactate Dehydrogenase, Lactic Acid, Swine, Troponin I

## Abstract

**Objective:**

To evaluate the effects of intracoronary iopromide (Ultravist®, Germany) administration on the recovery of cardiac pump function and cardiomyocytes metabolism during ex vivo cold preservation of pig hearts in the early posttransplant period.

**Methods:**

Three-month-old mini pigs weighing 73 ± 2.8 kg were used as experimental models (n=12). Physiological parameters were obtained with the IntelliVue MP70 system (Philips, Netherlands). Blood samples were taken from the coronary sinus to evaluate myocardial ischemia markers - troponin I, creatine phosphokinase-MB, lactate dehydrogenase, and lactate - and apex biopsy was performed before and after the ischemia period according to the protocol. Myocardial samples were taken from the left ventricle and prepared according to the protocol either.

**Results:**

Twelve orthotopic heart transplantations were performed during the study. Sample size was divided into two groups with six each. Cardiac output was 5.11 (4.99; 5.41) l/min and 5.77 (4.97; 6.62) l/min (P-0.0009) after 120 minutes of cardiac activity in both groups. Change of lactate dehydrogenase, creatine phosphokinase-MB, and troponin I levels in the coronary sinus blood were significantly higher in the early reperfusion period. However, there were no statistically significant differences between the groups (P>0.05). Myocardial oxygen consumption was considerably reduced during reperfusion but returned to baseline by 60 minutes of postischemia without significant differences between groups (P>0.05).

**Conclusion:**

We observed that intracoronary iopromide administration was safe during the ex vivo stage cold preservation phase of the study. Intracoronary iopromide administration did not affect cardiac pump function and cardiomyocytes metabolism in the early posttransplant period.

## INTRODUCTION

**Table:** 

Abbreviations, Acronyms & Symbols				
ATP	= Adenosine triphosphate		LV mass	= Left ventricular mass
CABG	= Coronary artery bypass grafting		LV O₂ cons.	= Left ventricular oxygen consumption
CAD	= Coronary artery disease		NO	= Nitric oxide
CAF	= Coronary artery flow		[O_2_]_a_	= Arterial blood oxygen level
CAV	= Cardiac allograft vasculopathy		[O_2_]_cs_	= Coronary sinus oxygen level
CO	= Cardiac output		O_2_ Sat	= Oxygen saturation
CPB	= Cardiopulmonary bypass		OCS	= Organ Care System
CPK-MB	= Creatine phosphokinase MB		OHT	= Orthotopic heart transplantation
Hb	= Hemoglobin		TnI	= Troponin I
HTK	= Histidine-tryptophan-ketoglutarate		VEGF	= Vascular endothelial growth factor
LDH	= Lactate dehydrogenase			

The demand for organ transplantation has led to extending donor criteria, with the age of donors increasing steadily for many years^[1,2]^. A recently published report (International Society for Heart & Lung Transplantation Thoracic Transplant Registry) demonstrated that the average age of donors increased from 31 to 35 years between 1992-2000 and 2010-2018^[3]^. However, older age is the most potent risk factor for coronary artery disease (CAD), which could increase mortality rate and early-onset cardiac allograft vasculopathy (CAV)^[4-6]^. In addition, younger recipients had a higher five-year survival rate^[3]^. In a multicenter study, Roig E. et al.^[6]^ included more than 2,000 patients from eight hospitals in Spain. They showed that the incidence of CAV onset was higher in patients over 50 years old and five years after the transplantation. Despite the fact that donor age is known to be an independent factor for higher recipient mortality^[7]^, the use of old donor hearts for old recipients remains unknown.

Due to the significant risk of CAD onset in elderly donors, the ability to evaluate coronary tree remains to be a unique technique for assessing the need for revascularization with coronary artery bypass grafting (CABG). According to the studies, stenosis of hemodynamic significance was discovered in 55% cases of coronary angiography in donors over 45 years old. In this connection, the study is recommended for donors over 40 years old or in case of doubt^[8-10]^.

However, coronary angiography is often unavailable for potential donors. The severity of the disease is a common reason for the inability to transport a donor to a better-equipped facility. It results in rejection to use an allograft with unknown coronary status or perform a transplantation with underestimated CAD and significant risk factors for posttransplant complications. *Ex vivo* coronary angiography is a possible option to avoid negative consequences.

*Ex vivo* coronary angiography is currently considered a diagnostic tool only when normothermic perfusion of the donor heart is facilitated by the TransMedics Organ Care System (OCS) from Massachusetts^[11,12]^. However, the widespread use of the Transmedics OCS is limited by its cost. The United Kingdom’s National Institute for Health reported that the cost of the OCS is close to 30,000 pounds^[9]^.

In fact, the first *ex vivo* coronary angiography was performed by Robicsek F. and Lee C.C. et al.^[13,14]^, in 1990-1992. Both authors showed that high-osmolar radiocontrast solutions did not reduce myocardial function when administered intracoronarily during the postischemic period. However, the design was not consistent with the standard clinical protocol for orthotopic heart transplantation, making it difficult to extrapolate the obtained data to the baseline protocol.

The aim of this study was to evaluate the effects of intracoronary administration of iopromide (Ultravist®, Germany) on the recovery of cardiac pump function and cardiomyocyte metabolism during the *ex vivo* preservation of cold donor hearts in the early post-transplant period.

## METHODS

### Ethical Statement

The study was approved by the local ethical committee of Meshalkin National Medical Research Center (Novosibirsk, Russia) and was conducted in accordance with the European Convention for the Protection of Vertebrate Animals Used for Experimental or Other Scientific Purposes (EST № 123 of 18.03.1986, Strasbourg). All animals were kept in standard vivarium conditions and had free access to water and feed.

### Design and Setting - Experimental Animals’ Preparation and Anesthesia

This research was carried out on three-month-old mini pigs. All animals received humane care in compliance with the Principles of Laboratory Animal Care formulated by the National Society for Medical Research and the Guide for the Care and Use of Laboratory Animals prepared by the Institute of Laboratory Animal Resources and published by the National Institutes of Health (NIH Publication No. 86-23, revised 1996).

**Fig. 1:**
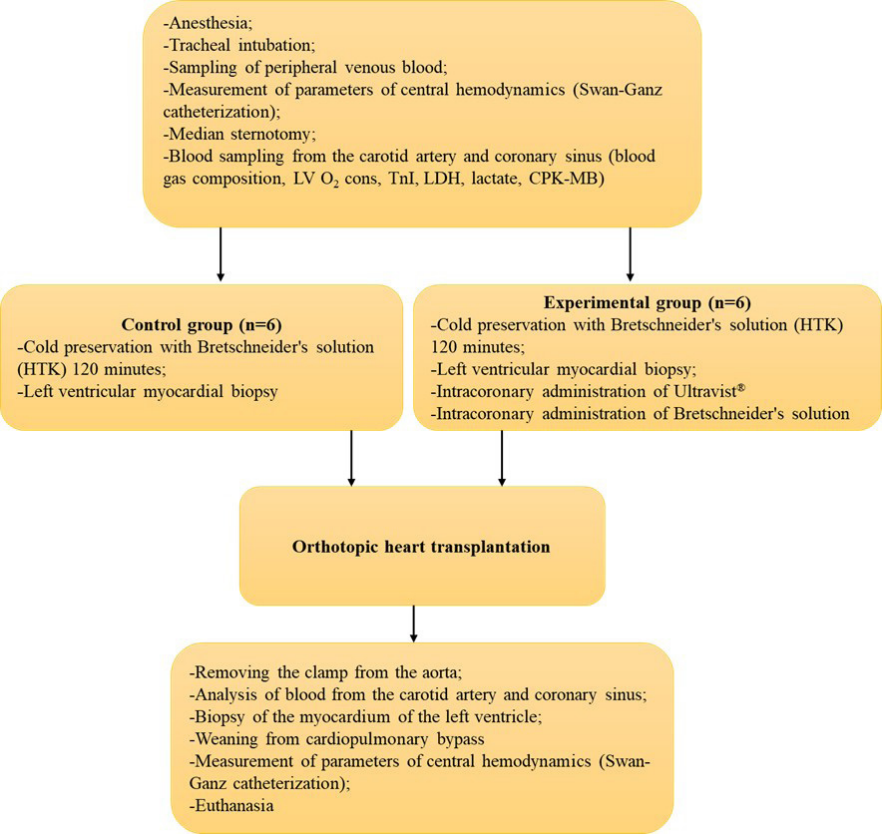
Study protocol. Physiological parameters were obtained with the IntelliVue MP70 system (Philips, Netherlands). Blood samples were taken from the coronary sinus to evaluate myocardial ischemia markers - troponin I (TnI), creatine phosphokinase MB (CPK-MB), lactate dehydrogenase (LDH), and lactate -, and apex biopsy was performed before and after the ischemia period according to the protocol. LV O_2_ cons.=left ventricular oxygen consumption; HTK=histidine-tryptophan-ketoglutarate.

Premedication was performed intramuscularly in the lateral part of the neck using atropine and Zoletil® 100 on the day of the surgery. Once sedated, the surgical field and the area for catheterization of neck vessels were prepared. The internal jugular vein and common carotid artery were cannulated for measurement of arterial and central venous blood pressure. General anesthesia was performed with sevoflurane and myorelaxation (pipecuronium bromide. The animals were connected to an automatic ventilator (Fabius® Plus, Dräger, Germany). Positive pressure for inspiration was set at 20-30 cm of water, while expiration was regulated at 5-8 cm of water. Additionally, a tidal volume of 8 ml/kg and a frequency of 12-14 breaths per minute were maintained. During the experiment, we controlled arterial blood pressure by cannulation of the left common carotid artery, central venous pressure by cannulation of the right external jugular vein, heart rhythm disturbances (electrocardiogram), body temperature, blood gas composition, and activated clotting time. Epicystostomy was performed to control diuresis. Blood analysis was performed by using a hematology analyzer XT-4000i (Sysmex, Germany) in accordance with recommendations. Central hemodynamics were obtained by Swan-Ganz catheterization of the right heart. Initial measurements were taken immediately after the start of endotracheal ventilation. Final measurements were taken two hours after weaning off cardiopulmonary bypass as recommended by the protocol (Figure 1).

### Surgical Techniques

#### Donor: Pig Heart Extraction and Cardioplegia

Donors with a higher body weight of 10-15 kg compared to matched recipients with an average weight of 86.3 ± 4.2 kg were premedicated and anesthetized as described above. The hearts were exposed by a median sternotomy in all cases. A cardioplegic 7 Fr cannula was placed in the aortic root after administration of heparin at a dose of 3 mg/kg. The ascending aorta was cross-clamped after occlusion of both caval veins, and cold cardioplegia was administered through the aortic root with two liters of Bretschneider’s histidine-tryptophan-ketoglutarate solution (Custodiol®, Germany). Hearts were stored in the appropriate solution at a temperature from 0 to 1°C for two hours. After two hours of conservation, non-selective angiography was performed by administrating iopromide (Ultravist®, Bayer, Germany) and HTK into the aortic root for five minutes under at a pressure of 70-80 mmHg in a 50:50 ratio. Afterwards, the coronary tree was washed out by one liter of HTK at a pressure of 40 mmHg. Consequently, the orthotopic heart transplantation was performed.

#### Recipient: Donor’s Heart Implantation

Hearts of mini pigs weighing 73 ± 2.8 kg were exposed by median sternotomy. After administration of heparin at a dose of 3 mg/kg, right common carotid artery and both caval veins were cannulated. CPB was then initiated, the donor heart was extracted with a wide cuff of pulmonary veins. Orthotopic transplantation of the donor heart was performed using bicaval technique: left atrium, pulmonary artery, aorta, and inferior and superior caval veins were subsequently anastomosed. For immunosuppression all recipients received pulse therapy with methylprednisolone (Methylpred® Orion, Portugal) at a dose of 1500 mg before removing the aortic clamp. The aorta was opened after taking precautions against air embolism. Heart reperfusion was started. Samples from the arterial cannula and coronary sinus were analyzed for myocardial oxygen consumption and markers of myocardial ischemia during the first minutes of reperfusion. After 30 minutes, a myocardial biopsy of the apex was performed, then myocardial defect was repaired with U-shaped sutures on felt pads. Finally, the recipients were gradually weaned off CPB. Comparative measurements were performed according to the protocol. Two hours later, recipients were euthanized by administration of 100 ml of 4% potassium chloride solution under general combined anesthesia (propofol [4-7 mg/kg], fentanyl [0.006-0.008 mg/kg], and inhalation of sevoflurane [2-4%]).

Myocardial samples were taken from the apex for histological examination. The samples were kept in 10% formalin on phosphate buffer (pH 7.4) and embedded in paraffin; they were sectioned using a microtome (5 µm) (Microm HM550). Hematoxylin and eosin staining was performed according to Van Gieson method, combining orcein for elastic fibers and periodic acid-Schiff reaction. General histological and morphometric studies were carried out using the micro-complex software with a light microscope (Carl Zeiss), an AxioCam MRc digital video camera, and Pentium 4 computer.

#### Preparation of Tissue Extracts

Myocardial samples were taken from the left ventricle. Then, the tissues were weighed, cut into small pieces, and added with 1 mL phosphate-buffered saline and stored at -70°C. The studied samples were homogenized using a KZ-III-FP Tissue Homogenizer Low-temperature (-40°C) (Servicebio Technology Co., Wuhan, China) with 3 mm*2/4 mm*1 steel balls as per the manufacturer’s recommendations. Tissue debris were removed by centrifugation at 16100 × g for five minutes. The contents of adenosine triphosphate (ATP), vascular endothelial growth factor (VEGF), nitric oxide (NO), and creatine kinase in the tissue extracts were normalized for protein concentration of each individual sample.

Commercially available enzyme-linked immunosorbent assay (or ELISA) kits were used to determine ATP (Cloud-Clone Corp., Wuhan, China) and VEGF (Vector-BEST, Novosibirsk, Russia) in tissue extracts from the left ventricle of the animal hearts. The kits were applied as per the manufacturer’s recommendations.

NO production was assessed by measuring the levels of nitrite as a stable end product using the Griess reagent (Sigma-Aldrich, Darmstadt, Germany) according to the manufacturer’s instructions. 50 µL of the tissue extracts were harvested and 50 µL of the Griess reagent were added to a 96-well plate. The absorbance at 492 nm was measured with a microplate reader (Stat FAX-2100, Awareness Technology Inc., United States of America), and the nitrite concentrations were estimated with a standard calibration curve.

### Statistical Analysis

Statistical analysis of this research was carried out using Statistica 10.0 software (StatSoft Inc., United States of America). All values are expressed as the mean ± standard deviation. Normal distribution was tested using Shapiro-Wilk test to contrast the hypothesis of the normality of the population scores. Further assessment was validated by Levene’s test. Student’s *t*-test was used according to group equivalence. Non-parametric methods were used in cases of abnormal statistical distribution. Statistical significance between the groups was established at *P*<0.05.

## RESULTS

**Fig. 2:**
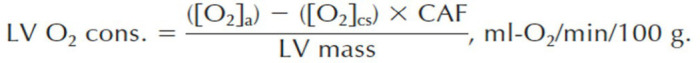
Myocardial oxygen consumption calculation. CAF=coronary artery flow; LV mass=left ventricular mass; LV O_2_ cons.=left ventricular oxygen consumption; [O_2_]_a_=arterial blood oxygen level; [O_2_]_cs_=coronary sinus oxygen level.

A total of 12 orthotopic heart transplantations were performed. The ischemia time of the donor heart was 184 ± 12 and 186 ± 10 minutes (*Р*>0.05) in experimental and control groups. The average time of the procedure was comparable between groups and was 44 ± 5 and 38 ± 8 minutes (*Р*>0.05). Non-selective coronary angiography allowed us to obtain high-quality images of coronary tree. Moreover, we determined with high accuracy the localization and severity of the simulated arterial stenosis (Figure 2).

Reperfusion time was 70 ± 8 minutes in all cases. Cardiotonic infusion was then started (dopamine 10 mcg/kg/min, adrenaline 0.1 mcg/kg/min), while pigs were gradually weaned off CPB in all cases. Changes in cardiac output (CO) were assessed at three different time points: 1) after weaning off CPB; 2) 60 minutes after weaning; and 3) 120 minutes after weaning. A statistically significant decrease in CO was observed in both groups compared to the baseline values (*Р*<0.05). However, the differences between both groups were insignificant (*P*>0.05) (Table 1).

**Table 1:** Cardiac output changes (l/min).

Group	Baseline	After CPB	After 60 min.	After 120 min.	*Р*-value
Control (n=6)	9.34 [9.15; 9.49]	6.35 [5.85; 6.91]	6.02 [5.82; 6.24]	5.11 [4.99; 5.41]	0.0009
Experimental (n=6)	9.47 [9.11; 9.91]	6.36 [5.92; 7.07]	7.04 [6.76; 7.17]	5.77 [4.97; 6.62]	0.0009

CPB=cardiopulmonary bypass

Lactate dehydrogenase (LDH), lactate, and troponin I (TnI) changes in coronary sinus blood were significantly higher in the early reperfusion period. However, no statistically significant differences were observed between the groups (*P*>0.05) (Table 2).

**Table 2:** Biochemical markers level changes in coronary sinus blood.

Measure/group	Control (n=6)		Experimental (n=6)	
	Before OHT	After OHT	Before OHT	After OHТ
LDH, U/l	1 429.85 [1279.4; 1540.325]	1 793.60^*^ [1388.1; 2079.625]	1 474.45 [1339.225; 1618.3]	1 719.25^*^ [1608; 1903.875]
TnI, ng/ml	14.80 [13.12; 16.1]	24 529.50^*^ [24353.02; 24634]	16.80 [11.1; 21.57]	23 754.55^*^ [23586.07; 23890.47]
Lactate, mmol/l	2.45 [2.2;2.9]	9.75^*^ [9.12; 0.75]	2.25 [1.97; 2.65]	9.05^*^ [8.2; 10.9]
CPK-MB, U/l	309.50 [212.5; 414.75]	285.50^#^ [264.5; 291]	309.50 [266.25; 386.75]	281.25^#^ [238.1; 332.6]

* *Р*<0.05 *vs.* concentration before OHT;

# *Р*>0.05 *vs.* concentration before OHT

CPK-MB=creatine phosphokinase MB; LDH=lactate dehydrogenase; OHT=orthotopic heart transplantation; TnI=troponin I

Myocardial oxygen consumption was significantly lower after reperfusion. However, it returned to its baseline value by 60 minutes without significant differences between the groups (*P*>0.05) (Table 3).

**Table 3:** Myocardial oxygen consumption (ml-О_2_/min/100g).

Group	Baseline	After unclamped	After reperfusion	*Р*-value
Control (n=6)	25.55 [24.2; 26.65]	1.685 [1.26; 2.27]	21.55 [19.95; 22.71]	0.0074
Experimental (n=6)	25.6 [24.8; 26.42]	1.23 [1.20; 1.32]	22.7 [21.42; 24]	0.0038

The concentrations of ATP and VEGF in myocardial samples were comparable in both groups. However, a significant decrease in NO concentration after heart transplantation was observed in the experimental group compared to the control group (Table 4).

**Table 4:** Changes in ATP, VEGF, and NO levels.

Measure/ group	Control (n=6)		Experimental (n=6)	
	Before OHT	After OHT	Before OHT	After OHТ
АТP, ng/ml (100 g/l protein conversion)	21.25 [17.77; 21.76]	17.07 [9.97; 20.26]^#^	21.13 [20.27; 21.96]	12.47 [9.93; 19.27]^#^
VEGF, pg/ml (100 g/l protein conversion)	26.28 [9.15; 27.31]	26.87 [18.69; 27.31]	21.24 [8.43; 35.8]	20.84 [19.07; 29.51]
NO, µM/mL (100 g/l protein conversion)	10.78 [8.26; 11.27]	11.31 [9.83; 11.59]^#^	12.84 [9.94; 13.35]	7.03 [5.76; 7.73]^* #^

* *Р*<0.05 *vs.* concentration after OHT control group;

# *Р*>0.05 *vs.* concentration before OHT

ATP=adenosine triphosphate; NO=nitric oxide; OHT=orthotopic heart transplantation; VEGF=vascular endothelial growth factor

**Fig. 3:**
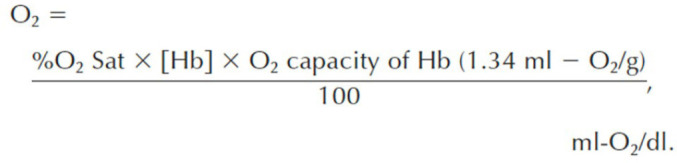
Blood oxygen level calculation. Hb=hemoglobin; O_2_ Sat=oxygen saturation.

Histological findings of myocardial parenchyma and stroma in experimental and control groups were generally similar. Muscle fibers of normal size and sarcoplasm of muscles were uniformly stained with hematoxylin and eosin (Figure 3). Transverse striations were clearly observed in the longitudinally sectioned fibers, as were areas of mild myofibril contracture.

**Fig. 4:**
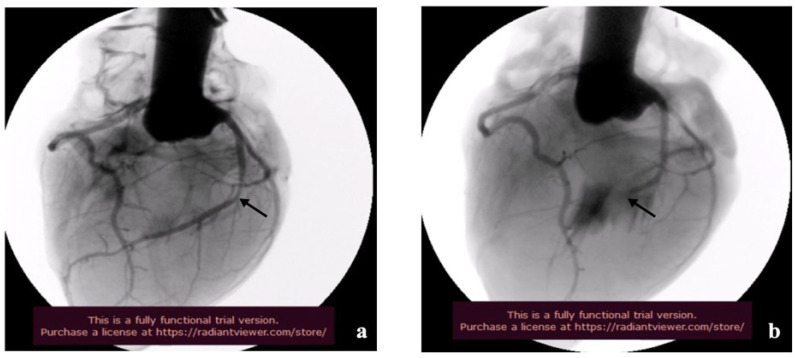
General view of non-selective coronary angiography: a) site of simulated anterior descending artery stenosis; b) site of the anterior descending artery occlusion (indicated by arrow) and the lack of the contrast below.

**Fig. 5:**
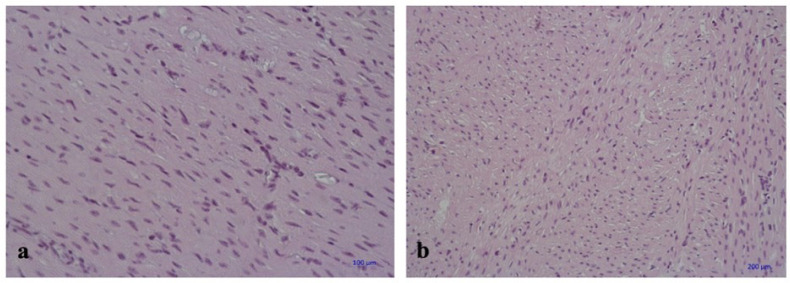
Myocardium of the left ventricle with the preservation of muscle fibers diameter and accurate fibers boundary. a) control group, hematoxylin-eosin stain, magnification × 400; b) experimental group, hematoxylin-eosin stain, magnification × 200.

**Fig 6:**
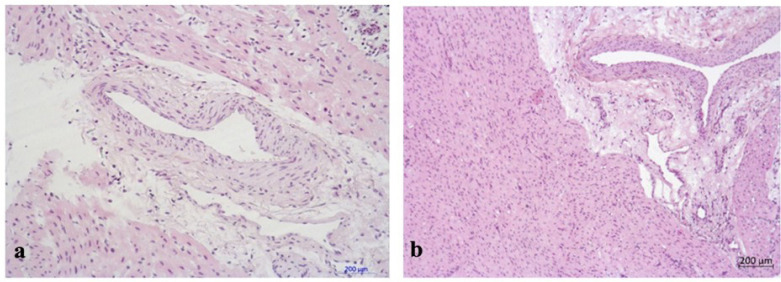
Epicardial vessels of the left ventricle with dilated lumen and preserved endothelial lining. a) control group, hematoxylin-eosin stain, magnification × 400; b) experimental group, hematoxylin-eosin stain, magnification × 200.

The epicardial stroma was moderately and patchily edematous. Medium and large caliber arteries were dilated. Endothelial cells were evenly divided, flat, and maintained their integrity. Numerous capillaries were observed within the myocardial stroma in fiber border space mainly with unaltered and dilated lumen, thin wall, and preserved endothelial lining (Figures 4, 5, and 6).

## DISCUSSION

Nowadays, the number of donors with non-traumatic injuries is increasing dramatically. In general, donors are older and have more comorbidities^[15,16]^. Considering that, one of the major causes of death among recipients within the first three years is concentric intimal proliferation of the coronary arteries in the posttransplant period. Therefore, the assessment of CAD is essential for predicting the graft dysfunction and the tactics of postoperative period for the recipients who received compromised grafts^[17-19]^. According to Eurotransplant, the survival rate of the recipients with intact coronary tree within the first three years after the procedure was 81%, compared with 50% who received a heart with documented coronary pathology^[20]^. Early studies demonstrated increasing of donor age was a risk factor for posttransplant CAD and/or worse prognosis^[21-25]^. Grauhan O. et al.^[26]^ showed that myocardial contractility of donor heart with verified coronary tree pathology did not differ significantly from that of intact donor heart. This is true prior reperfusion when angiographic evidence of CAD is detected to perform CABG^[27]^. Pozniak M.A. et al.^[27]^ confirmed that survival rate was lower in the early posttransplant period in the donors aged 40 years or over with unknown coronary status. However, routine selective coronary angiography is technically and logistically difficult to perform. For this reason, development of a safe and effective method of preventive coronary angiography of the donor heart *ex vivo* remains a major problem^[29]^.

The administration of contrast solution during preservation is potentially dangerous for the allograft due to intentional violation of the cold preservation protocol. However, the study showed the safety of intracoronary drug administration, despite excessive exposure of the Ultravist® solution. We did not find any significant differences in the recovery of heart pump function between the groups. The cause of the decrease in CO after weaning off CPB was the large mass of the donor heart and low efficiency of standard cold preservation protocol, which is indicated by increasing levels of TnI, LDH, and lactate during reperfusion in both experiments.

Studies on the intra-arterial use of X-ray contrast agents indicate that they may reduce capillary blood flow, leading to a decrease in tissue oxygen partial pressure. In our investigation of iopromide (Ultravist®) on human endothelial cell morphology in vitro, we observed a 95% increase in the height of endothelial cells compared to the control group (*P*=0.0065). This could potentially result in a threefold decrease in the partial pressure of oxygen in the relevant artery's tissue for 50% of cases following the *in vivo* bolus administration of iopromide into the left coronary artery^[28,29]^. We demonstrated a sharp drop in myocardial oxygen consumption in average of 1.2 ± 0.2 ml-О_2_/min/100g during the first 10-15 minutes of reperfusion with subsequent improvement of parameters for 40-50 minutes without significant difference between both groups.

Pathomorphological examination did not show considerable histological differences in samples of both groups. All specimens showed moderate stromal edema and isolated cardiomyocytes with no evidence of damage to the endothelial myocardial capillary layer.

### Limitations

This study has several limitations. Firstly, the small number of animals involved could be seen as a constraint; however, this did not hinder the achievement of statistically significant results, highlighting the existing disparities. Although the mini pigs were matched for SLA class I antigen and sourced from the same litter, some degree of biological variation among the animals was inevitable and may have influenced the final outcomes. Additionally, in this experimental model of heart transplantation, Bretschneider’s HTK solution (Custodiol®, Germany) was used as a preservation solution, but the individual sensitivity of the preservative on the hearts and the technique itself were not investigated.

## CONCLUSION

The experiment validated the high diagnostic value of non-selective coronary angiography in pig hearts and established the safety of intracoronary administration of iopromide during *ex vivo* cold heart preservation. Furthermore, the administration of intracoronary iopromide did not affect the recovery of pump function or the metabolism of cardiomyocytes in the early post-transplant period.
